# Supplementary figures and images for: Demonstrating the utility of flexible sequence queries against indexed short reads with FlexTyper
Source: PLoS Comput Biol. 2021 Mar 22;17(3):e1008815. doi: 10.1371/journal.pcbi.1008815 (PMC8016220; doi:10.1371/journal.pcbi.1008815)

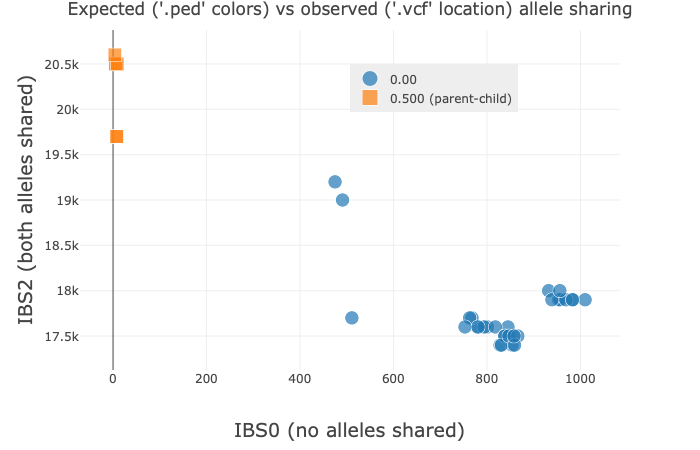

Supplement: S1 Fig — Relatedness comparison for the three trios correctly identifies the six relationships of parent-offspring (orange squares) and correctly identifies a lack of relatedness for the other comparisons. (TIF) [file pcbi.1008815.s001.tif]
