# Supplementary material for: Demonstrating the utility of flexible sequence queries against indexed short reads with FlexTyper
Source: PLoS Comput Biol. 2021 Mar 22;17(3):e1008815. doi: 10.1371/journal.pcbi.1008815 (PMC8016220; doi:10.1371/journal.pcbi.1008815)
Supplement: S1 Supplemental Methods — (DOCX) [file pcbi.1008815.s006.docx]

# **S1 Supplemental Methods**

For the manuscript:

“Demonstrating the utility of flexible sequence queries against indexed short reads with FlexTyper”

Richmond & Kaye et al.

### Data sources for query generation

CytoScanHD SNP probe set was created using the file CytoScanHD_Array.na33.annot.csv acquired from the Affymetrix site (<https://www.thermofisher.com/ca/en/home/life-science/microarray-analysis.html>). This file was processed using CytoscanSProbe2Query.py (<https://github.com/wassermanlab/OpenFlexTyper>).

GRCh37 ancestry sites were acquired from <https://github.com/brentp/peddy/blob/master/peddy/GRCH37.sites>. These sites were then converted into a query file using Sites2Query.py.

Pathogen fasta sequences were acquired from NCBI for EBV (gi|82503188|ref|NC_007605.1| Human gammaherpesvirus 4), HIV-1 (gi|4558520|gb|AF033819.3| HIV-1), U21941.1 (U21941.1 Human papillomavirus type 70), and FR751039.1 (FR751039.1 Human papillomavirus type 68b). Each of these fasta files was then converted into a query file using the fasta_2_query.py script (<https://github.com/wassermanlab/OpenFlexTyper/tree/master/fmformatter>).

African contigs were pulled from NCBI (<https://www.ncbi.nlm.nih.gov/nuccore/PDBU01000000>), and converted into query files using fasta_2_query.py.

KIR sequence kmers were downloaded from <https://github.com/droe-nmdp/kpi in September 2020>, and used directly with the kmer searching tool after extracting sequence and identifier columns.

### Samples for genotype analysis testing

To demonstrate the genotyping capacity of FlexTyper at known SNP sites, we used WGS datasets from the Polaris project Diversity Cohort and Kids Cohort (<https://github.com/Illumina/Polaris>). These WGS data sets were downloaded as raw fastqs, mapped against GRCh37 genome using BWA mem (v0.7.5), and then converted to BAM format using Samtools (v1.9). Variants were then called using DeepVariant (v0.10.0) [1, 2]. All WGS processing for fastq → vcf  was done on an HPC cluster scheduler using a maximum of 150GB of RAM with 32 CPUs. We additionally tested the indexing capacity using sample HG002, downloaded from <https://ftp-trace.ncbi.nlm.nih.gov/ReferenceSamples/giab/data/AshkenazimTrio/HG002_NA24385_son/NIST_Illumina_2x250bps/reads/>.

### Simulation of pathogen-containing samples

To simulate pathogen samples in RNA-seq data, we first acquired fasta sequences for four different viruses (described above) and simulated reads at various depths of coverage for paired-end 150bp reads using the read simulator ART [3]. We tested the recovery of these reads on the simulated files, and additionally spiked them into RNAseq samples. The human RNA-seq samples come from the Genome England project (<https://www.ebi.ac.uk/arrayexpress/experiments/E-MTAB-6523/samples/>). We chose five different human blood RNA-seq datasets to mix with differing read counts of each virus, as defined in Table 1. The patients use blood derived from sample accessions as follows: Patient_1-ERR2322363, Patient_2:ERR2322364, Patient_3:ERR2322365, Patient_4:ERR2322366, Patient_5:ERR2322363.

### BAM coverage calculation for comparison

Bam coverage is calculated from an input BAM file and a FlexTyper query file using the script QueryFromBam.py (<https://github.com/wassermanlab/OpenFlexTyper/tree/master/extras/bamquery>). This script utilizes the Pysam package to extract the reference and alternate alleles present at a given locus as defined from an input FlexTyper query file.

### Using Peddy for ancestry, sex, and relatedness typing

After running FlexTyper on the ancestry + chrom X sites, we converted the output format to VCF for input into Peddy. The resulting VCF files for all nine individuals were then compressed with bgzip and indexed with tabix (v1.9), and then merged with bcftools merge (v1.10.1). Next, a PED file was created detailing the relationships and sex of each individual based on information from the Polaris data repository (<https://github.com/Illumina/Polaris>). We used the merged VCF and the PED file as input to Peddy (v0.4.3) and ran with default settings to generate ancestry, relatedness, and sex-typing figures [4].

### Running Centrifuge and Kraken2 on simulated patient data

Centrifuge (v1.0.4) was run with default parameters on the simulated “patient” fastqs, where each patient had mixed viral and human blood RNA-seq reads. The output report files from centrifuge were parsed for the viral names using grep: Human immunodeficiency virus 1 (HIV-1), Human herpesvirus 4 type 2 (EBV), and “apilloma” to get all strains of Human (and other) Papilloma viruses (HPV). The numReads (number of reads) were used as a fair comparator to FlexTyper which also was using non-unique read counting for viral detection.

Kraken2 (2.0.9) was run with default parameters on the standard parameters and outputting the mpa-style format, on the default database for human + viral + bacterial genomes (built on June 30th 2020). For collating counts from the mpa-style output report file, with EBV and HIV1 we select the value at the species level as these were accurately assigned. For collating counts from U21941 and FR751039, we extracted counts at the family (Papillomaviridae) level. For subsequent analysis of pure viral reads, we ran Kraken2 independently on 5200 simulated reads from each of the two genomes and collated the output in the same fine.

### Using FlexTyper: Parameter explanation

**Sliding Search Parameters**


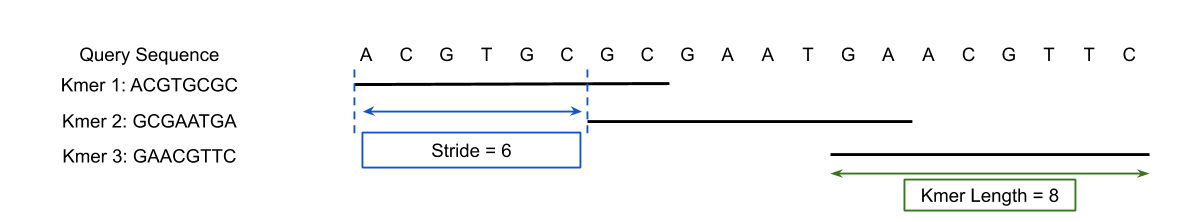


**Centered Search Parameters**


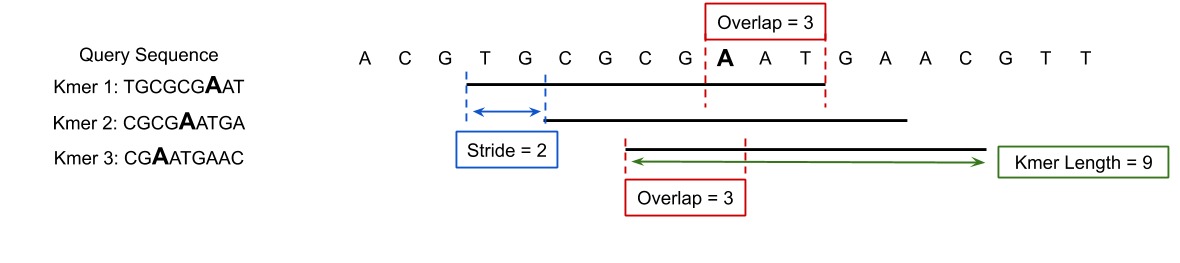


**Non Unique k-mers**

If two query sequences contain an identical k-mer, then that k-mer is flagged as a ‘non unique k-mer’.


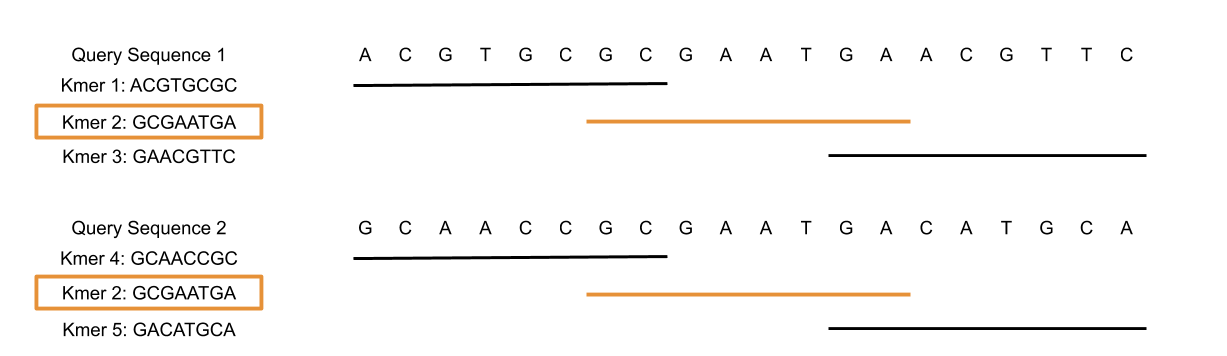


**Over Counted k-mers**

If a k-mer search returns too many positions, then that k-mer is disregarded as uninformative and flagged as an “over counted k-mer”.

**Count As Pairs**

For paired reads [R1 = <1,1>, R2 = <1,2>]

If countAsPairs = true, then if a query matches R1 and R2, then the query count is only one.

**Unique Reads**

For two queries Q1 and Q2, uniqueReads = true, then if a read, R, matches Q1 and Q2, then R will be removed from the set of matching read hits for both Q1 and Q2.

**Combining countAsPairs and uniqueReads**

Suppose you have 3 sets of paired reads:
Pair 1: [R1 = <1,1>, R2 = <1,2>]

Pair 2: [R3 = <2,1>, R4 = <2,2>]

Pair 3: [R5 = <3,1>, R6 = <3,2>]

And two query sequences with matching read hits:
Query 1 = [R1, R3, R5, R6]

Query 2 = [R1, R4]

|  | countAsPairs = True | | | countAsPairs = False | | |
| --- | --- | --- | --- | --- | --- | --- |
|  | Query | Read Hits | Count | Query | Read Hits | Count |
| uniqueReads = True | 1 | [R5, R6] | 1 | 1 | [R3, R5, R6] | 3 |
|  | 2 | [ ] | 0 | 2 | [R4] | 1 |
| uniqueReads = False | 1 | [R1, R3, R5, R6] | 3 | 1 | [R1, R3, R5, R6] | 4 |
|  | 2 | [R1, R4] | 2 | 2 | [R1, R4] | 2 |

# **References**

1. Li H, Durbin R. Fast and accurate short read alignment with Burrows-Wheeler transform. Bioinformatics. 2009;25(14):1754-60. doi: 10.1093/bioinformatics/btp324. PubMed Central PMCID: PMCPMC2705234.

2. Li H, Handsaker B, Wysoker A, Fennell T, Ruan J, Homer N, et al. The Sequence Alignment/Map format and SAMtools. Bioinformatics. 2009;25(16):2078-9. doi: 10.1093/bioinformatics/btp352.

3. Huang W, Li L, Myers JR, Marth GT. ART: a next-generation sequencing read simulator. Bioinformatics. 2012;28(4):593-4. doi: 10.1093/bioinformatics/btr708. PubMed Central PMCID: PMCPMC3278762.

4. Pedersen BS, Quinlan AR. Who's Who? Detecting and Resolving Sample Anomalies in Human DNA Sequencing Studies with Peddy. Am J Hum Genet. 2017;100(3):406-13. doi: 10.1016/j.ajhg.2017.01.017. PubMed Central PMCID: PMCPMC5339084.
